# Interaction between glycolipid MPIase and proteinaceous factors during protein integration into the cytoplasmic membrane of *E. coli*

**DOI:** 10.3389/fmolb.2022.986602

**Published:** 2022-08-19

**Authors:** Hanako Nishikawa, Katsuhiro Sawasato, Shoko Mori, Kohki Fujikawa, Kaoru Nomura, Keiko Shimamoto, Ken-Ichi Nishiyama

**Affiliations:** ^1^ Department of Biological Chemistry and Food Science, Faculty of Agriculture, Iwate University, Morioka, Japan; ^2^ Bioorganic Research Institute, Suntory Foundation for Life Sciences, Kyoto, Japan

**Keywords:** membrane protein insertion, glycolipid, MPIase, SecYEG, YidC, proton motive force

## Abstract

Protein integration into biomembranes is an essential biological phenomenon common to all organisms. While various factors involved in protein integration, such as SRP, SecYEG and YidC, are proteinaceous, we identified a glycolipid named MPIase (Membrane Protein Integrase), which is present in the cytoplasmic membrane of *E. coli*. *In vitro* experiments using inverted membrane vesicles prepared from MPIase-depleted strains, and liposomes containing MPIase showed that MPIase is required for insertion of a subset of membrane proteins, which has been thought to be SecYEG-independent and YidC-dependent. Also, SecYEG-dependent substrate membrane proteins require MPIase in addition. Furthermore, MPIase is also essential for insertion of proteins with multiple negative charges, which requires both YidC and the proton motive force (PMF). MPIase directly interacts with SecYEG and YidC on the membrane. MPIase not only cooperates with these factors but also has a molecular chaperone-like function specific to the substrate membrane proteins through direct interaction with the glycan chain. Thus, MPIase catalyzes membrane insertion by accepting nascent membrane proteins on the membrane through its chaperone-like function, i.e., direct interaction with the substrate proteins, and then MPIase functionally interacts with SecYEG and YidC for substrate delivery, and acts with PMF to facilitate and complete membrane insertion when necessary. In this review, we will outline the mechanisms underlying membrane insertion catalyzed by MPIase, which cooperates with proteinaceous factors and PMF.

## Membrane protein integration and presecretory protein translocation

**FIGURE 1:**
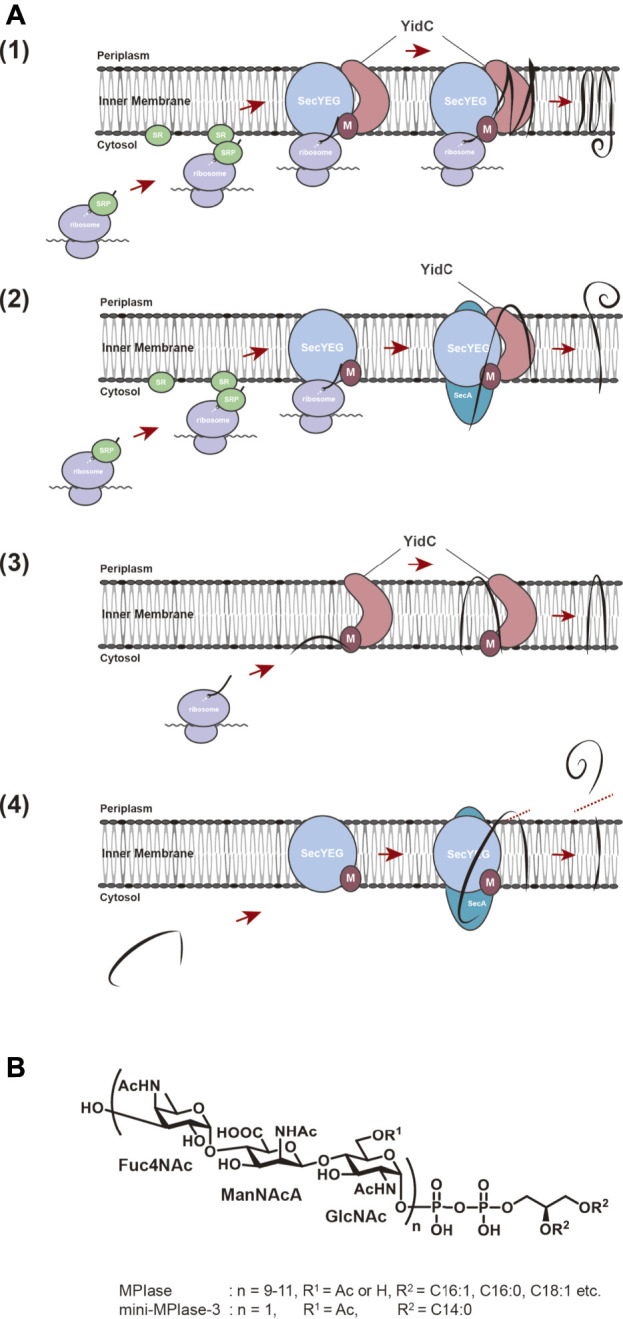
**(A)** Membrane insertion pathway for proteins in *E. coli*. (1) Sec-dependent membrane insertion pathway. (2) SecYEG/SecA-dependent membrane insertion pathway. (3) Sec-independent membrane insertion pathway. (4) Sec-dependent preprotein translocation pathway. Black lines represent substrate proteins. **(B)** Structures of MPIase and chemically synthesized mini-MPIase-3. The glycan chain of MPIase is bound to DAG via pyrophosphate. The glycan consists of a repeating sugar chain composed of a trisaccharide unit of 4-acetamido-4-deoxyfucose (Fuc4NAc), 2-acetamido-2-deoxymannuronic acid (ManNAcA), and *N*-acetyl-glucosamine (GlcNAc). While the number of repeats (n) is 9–11 in MPIase, chemically synthesized mini-MPIase-3 has only one trisaccharide unit. R^1^ is H or Ac in MPIase, and Ac in mini-MPIase-3. R^2^ represents fatty acids C16:1, C:16:0, C18:1, C18:0 in MPIase, and C14:0 in mini-MPIase-3, respectively.

Proteins destined to be embodied into the biological membrane or to be exported across the membrane require a specific mechanism to reach the destination to exert their function at the proper location. The molecular mechanisms of these processes commonly occur in all living organisms, from bacteria to higher eukaryotes. The outstanding discovery in this field was done by Blobel in 1975. His studies on membrane translocation of presecretory proteins in the endoplasmic reticulum (ER) led to the proposal of the “signal hypothesis” to explain how proteins reach their final destinations (Blobel and Dobberstein, 1975). Presecretory proteins possess an extended sequence, the so-called signal sequence, of 20–40 amino acids at their N-termini, which serves as a “tag” for the translocation of precursor proteins to their destinations. When presecretory protein synthesis is initiated and the signal sequence gets exposed on ribosomes, the hydrophobic region of the signal sequence is recognized by the signal recognition particle (SRP) and directed onto the ER *via* the SRP receptor (SR) on the membrane (Walter and Blobel, 1981a; Walter and Blobel, 1981b; Walter et al., 1981). Ribosomes-nascent chain complex (ribosomes with nascent polypeptides emerging from the ribosomes) then interacts with the protein-conducting channel (the Sec61 complex) on the ER membrane and deliver the nascent chains of precursor proteins into the channel co-translationally (Zito and Oliver, 2003). After insertion into the ER membrane, the signal sequence is cleaved off by a signal peptidase to give a mature protein. The “signal hypothesis” can explain the molecular mechanism of membrane integration of hydrophobic membrane proteins. The hydrophobic transmembrane (TM) regions of nascent chain of a membrane protein are recognized by SRP through the interaction with the TM regions, the protein then being transported to the ER membrane via the SR. The process prevents aggregation of hydrophobic TM domain of nascent protein in the hydrophilic environment of cytoplasm. Then, integration proceeds on the protein conducting channels (SecYEG in bacteria and the Sec61 complex in eukaryotes) co-translationally (Sec-dependent membrane integration pathway) (Rapoport et al., 2004; Luirink et al., 2005; du Plessis et al., 2011) (Figures 1A1). In *E. coli*, membrane proteins with large hydrophilic regions on the periplasmic side require SecA, a motor protein with ATPase activity, in addition (Andersson and von Heijne, 1993; Neumann-Haefelin et al., 2000) (Figure 1A2). Membrane proteins of small molecular weight or ones with only a TM domain at the very C-terminus are independent of SRP/SR and SecYEG for membrane insertion (Sec-independent membrane insertion pathway) (Andersson and von Heijne, 1993; Sargent et al., 1999) (Figure 1A3), because their synthesis ceases before the nascent chain interacts with SRP. Sec-independent insertion has long been thought to proceed spontaneously through hydrophobic interactions between phospholipids forming the membrane bilayers and the TM regions of membrane proteins (Geller and Wickner, 1985; Kuhn, 1995; Kiefer and Kuhn, 1999). On the other hand, the observations that the membrane insertion of several Sec-independent substrate proteins is inhibited by YidC depletion (Samuelson et al., 2000), and that YidC directly interacts with SecYEG (Petriman et al., 2018) led to the proposal that YidC is a “membrane protein insertase” that catalyzes membrane insertion reactions (Serek et al., 2004). Furthermore, YidC has been reported to function as a molecular chaperone that is involved in folding of polytopic proteins in the membrane, which are inserted in a Sec-dependent manner (Nagamori et al., 2004).

## Blockage of spontaneous insertion by diacylglycerol

An *in vitro* reconstitution system of the membrane protein insertion reaction has been used as an effective approach to analyze the molecular mechanisms underlying protein insertion in detail. A SecYEG- and SRP/SR-dependent substrate, mannitol permease (MtlA), was found to be spontaneously inserted into liposomes formed with only phospholipids (Nishiyama et al., 2006). Since such disordered spontaneous insertion is unlikely to occur *in vivo*, we considered that a system by which the spontaneous insertion is blocked is present in biomembranes. Therefore, it was necessary to clarify the system and to determine the compounds necessary for it to verify the insertion-factor dependencies for protein insertion. A search for compounds involved in the blockage of spontaneous integration revealed that the addition of DAG, a component of the cytoplasmic membrane of *E. coli*, at physiological concentrations completely blocked the spontaneous insertion of MtlA (Nishiyama et al., 2006; Kawashima et al., 2008). In the presence of DAG, spontaneous insertion of not only Sec-independent Pf3 coat and M13 procoat but also 3L-Pf3 coat, a mutant of Pf3 coat, which was thought to be inserted independently of any proteinaceous factor or membrane potential (Kiefer and Kuhn, 1999; Serek et al., 2004), was completely blocked (Kawashima et al., 2008). DAG is known to enter the gaps between phospholipids inside the lipid bilayer, thereby tightening the packing of the lipid bilayer and preventing lateral diffusion of membrane lipids (Schorn and Marsh, 1996; Alwarawrah et al., 2012). Thus, it is assumed that DAG prevents spontaneous integration of membrane proteins due to these properties (Nomura et al., 2019). Addition of DAG to liposomes allowed us to develop a reconstitution system of membrane protein insertion that faithfully reflects the *in vivo* situation (Kawashima et al., 2008).

## Discovery of membrane protein integrase

Under the conditions in which spontaneous insertion had been completely blocked, even substrates that were previously thought to be inserted spontaneously failed to be insert into liposomes, strongly suggesting that membrane insertion of these substrates is dependent on an unknown insertion factor (s). A search for this factor led to identification of a compound of approximately 7 kDa on SDS-PAGE (Nishiyama et al., 2010). By means of a reconstitution system involving (proteo)liposomes containing DAG to block spontaneous insertion, in which this factor had been included, we found that not only Sec-dependent MtlA but also Sec-independent M13 procoat was inserted in a factor-dependent manner (Nishiyama et al., 2006), indicating that this factor is involved in the overall membrane insertion/integration of substrate proteins, irrespective of it being Sec-dependent or Sec-independent. When the amount of substrate protein in the reaction mixture was increased, the amount of membrane-inserted protein was greater than the amount of this factor, indicating that this factor can drive multiple insertion cycles (Nishiyama et al., 2010). Surprisingly, this factor turned out to be not a protein but a glycolipid (Nishiyama et al., 2012). Based on these observations, we proposed the concept of a “glycolipozyme,” because this factor exhibits enzyme-like properties, catalyzing membrane insertion reactions, even though it is a glycolipid. Therefore, we named this factor MPIase (membrane protein integrase) after its enzyme-like properties (Nishiyama et al., 2010; Nishiyama et al., 2012).

## Relationship between the structure and function of membrane protein integrase

The structure of MPIase was determined by means of NMR and MS analyses of purified and chemically synthesized MPIase preparations (Nishiyama et al., 2012; Fujikawa et al., 2018) (Figure 1B). Gel filtration analysis using an MPIase derivative (PP-MPIase), in which MPIase is cleaved at the center of pyrophosphate to form a soluble sugar chain, suggested that MPIase forms an oligomer (Nishiyama et al., 2012). Since all the sugars constituting the trisaccharide unit of MPIase have *N*-acetyl groups and about 30% of GlcNAc 6-OH is also acetylated, a large number of acetyl groups is present on MPIase. Structure-function analysis using chemically synthesized mini-MPIase-3 (with only one trisaccharide unit) revealed that this structure contains the minimum unit with insertion activity and that the acetyl groups on GlcNAc play an important role in membrane insertion (Fujikawa et al., 2018). Using chemically synthesized MPIase derivatives, we investigated the function of each MPIase substructure in membrane insertion. We found that the lipid moiety and phosphate group of MPIase are also essential for the activity (Fujikawa et al., 2018). The lipid moiety may be necessary to anchor MPIase on the membrane surface, and to capture hydrophobic substrate proteins on the membrane and deliver them into the membrane. In this case, MPIase may act cooperatively with ribosomes and other membrane insertion factors. Also, it was proved that PP-MPIase directly interacts with substrate proteins through numerous acetyl residues to form a soluble complex, preventing aggregation (Nishiyama et al., 2012). Thus, the sugar chain of MPIase has a molecular chaperone-like function that prevents aggregation of substrate proteins. Phosphate groups are involved in this interaction as well, where the negative charges of the phosphate groups are thought to interact with the positive charges of the substrate proteins (Breukink et al., 1992; Crooke, 2001; Matsumoto, 2001). On the other hand, the insertion activity decreased as the sugar chain length became shorter, indicating that a sufficient sugar chain length is essential for preventing aggregation of the substrate proteins (Fujikawa et al., 2018).

Recently, an enzyme that is involved in the first step of MPIase biosynthesis was discovered, and then the MPIase-depleted strains have been constructed (Sawasato et al., 2019). Under MPIase-depleted conditions, membrane insertion of M13 procoat is completely abolished and the rate of translocation of pOmpA is significantly retarded. With the marked effects on protein transport, MPIase-depleted strains are lethal, indicating that MPIase is essential for cell growth (Sawasato et al., 2019). Thus, MPIase, identified by biochemical analysis, proved to have the expected functions *in vivo* as well.

## Interaction between membrane protein integrase and YidC

YidC was proposed to be an insertase that catalyzes Sec-independent protein membrane insertion (Samuelson et al., 2000; Serek et al., 2004). Membrane insertion of Sec-independent substrate M13 procoat was completely inhibited in terms of inhibition of processing N-terminal signal sequence which occurs after transmembrane domains span the membrane under the YidC-depleted conditions, however, a precursor protein could be targeted to the membrane, and it was resistant to alkaline extraction. These observations suggest that protein insertion proceeds to some extent even if YidC is absent (Samuelson et al., 2001). Both *in vivo* analyses using MPIase-depleted strains and *in vitro* reconstitution studies with DAG revealed that M13 procoat and Pf3 coat, of which insertion was thought to be dependent on YidC, were not inserted into membranes with YidC alone, but were into ones with MPIase (Sawasato et al., 2019). These findings suggest that the role of YidC in membrane integration should be reconsidered. Analyses of the Sec-independent insertion have led to many different conclusions, which have hampered a unified understanding of the relationship between MPIase and YidC, and of their respective functions. To overcome these controversial and reveal precise role of YidC, we set out to develop *in vitro* system that faithfully reproduce *in vivo* reaction. A Sec-independent substrate, *E. coli* F_0_c (the c subunit of F_0_F_1_-ATPase), was reported to be YidC-dependent for insertion *in vivo* (Samuelson et al., 2001) and *in vitro* (van der Laan et al., 2004). On the other hand, there have been many contradictory reports, such as that YidC is not required for insertion in the reconstitution system (Robinson and Woolhead, 2013) and that F_0_c insertion occurs spontaneously even when DAG is included in liposomes (Robinson and Woolhead, 2013). By mean of a reconstitution system we developed, we have clarified that MPIase is essential for F_0_c insertion (Nishikawa et al., 2017). Further, we demonstrated that although YidC alone is not sufficient for the insertion, YidC significantly stimulated the MPIase-dependent insertion of F_0_c (Nishikawa et al., 2017). Based on the crystal structure of YidC (Kumazaki et al., 2014a), a model for membrane insertion has been proposed. YidC possesses a hydrophilic “groove” with an arginine residue at its center in the cytoplasmic membrane. The positive charge of the arginine residue attracts the negatively charged regions of the substrate protein through electrostatic interaction, which results in its incorporation into the membrane (Kumazaki et al., 2014a). This model is consistent with that MPIase functions at the initial stage of the insertion reaction on the cytoplasmic surface of the membranes, and then YidC receives a partially inserted substrate through the electrostatic interaction to promote and complete insertion. This model predicts the presence of a functional and cooperative interaction between MPIase and YidC (Nishikawa et al., 2017). Furthermore, this study demonstrated that MPIase also plays a role in inhibiting the formation of protease-resistant conformations of F_0_c (Nishikawa et al., 2017). This function is consistent with the molecular chaperone-like properties of MPIase dedicated to the membrane proteins (Nishiyama et al., 2012; Fujikawa et al., 2018).

The interplay of MPIase with YidC and PMF in the Sec-independent pathway has also been analyzed using different substrates (Endo et al., 2022). *In vivo* experiments have shown that Pf3-Lep, an N-out type substrate, requires neither YidC nor PMF for membrane insertion, while its mutant, V15D, with increased N-terminal negative charges requires both YidC and PMF (Zhu et al., 2013). A reconstitution system was constructed and the dependency of each substrate on the membrane insertion factors *in vitro* was verified. MPIase was required for insertion of both substrates. In the case of Pf3-Lep, the dependency on YidC/PMF increased with increasing substrate level, whereas V15D insertion was promoted by YidC/PMF regardless of the substrate level (Endo et al., 2022). These results indicate that the number of negative charges on the N-terminal side and the substrate level strongly affect the dependency on YidC and PMF. These results are consistent with the observations that electrostatic interaction between YidC and substrate proteins, and translocation of the negatively charged residues of the substrate to the periplasmic side are facilitated by PMF (Kumazaki et al., 2014a; 2014b). The change in the factor dependency caused by an increased substrate level has also been observed for the Sec/YidC/PMF-independent substrate, 3L-Pf3 coat (Sasaki et al., 2019) and Sec-dependent MtlA (Sasaki et al., 2019), as well as for Pf3-Lep (Endo et al., 2022). MPIase is essential for membrane insertion of all the substrates regardless of the substrate amount, but when the substrate amount is low, the insertion of the substrates is not dependent on YidC. As the substrate level increases, the insertion becomes YidC-dependent. These results indicate that the interplay of three factors, MPIase, YidC and PMF, is important for efficient protein insertion (Endo et al., 2022). A functional MPIase-YidC interaction is also suggested *in vivo*, since the expression level of MPIase remarkably increases under YidC-depleted conditions. Consistently, direct interaction between MPIase and YidC has been demonstrated by pull-down assays (Endo et al., 2022).

In addition to the function related to membrane insertion, YidC has been reported to be involved in the folding of membrane proteins (Nagamori et al., 2004). We also attempted to reconstitute a series of reactions from membrane insertion to folding and oligomerization using F_0_c of *Propionigenium modestum* (Pm-F_0_c) (Suzuki et al., 2007; Ozaki et al., 2008), which, like *E. coli* F_0_c, forms a complex (11 dimer in the case of Pm-F_0_c) with a ring structure after membrane insertion. We found that membrane insertion of Pm-F_0_c is MPIase-dependent and that YidC functions at a late stage of insertion (Nishikawa et al., 2021). Furthermore, YidC is involved in the formation of a c_11_ ring structure by cooperatively interacting with Pm-UncI, which is essential for Pm-F_0_c ring formation (Nishikawa et al., 2021). Based on these results, it is highly likely that YidC interacts with MPIase at the stage of membrane insertion, and then with UncI in the subsequent stages of folding and oligomerization.

## Interaction between membrane protein integrase and SecYEG

The cooperative interaction of MPIase with membrane integration factors also occurs in the Sec-dependent pathway. It is reported that either SecYEG or YidC is sufficient for membrane integration of MtlA, a Sec-dependent substrate (Welte et al., 2012). In this study, however, since removal of the detergent during proteoliposome preparation was insufficient (Sasaki et al., 2019), MtlA integration might not have been properly reproduced, causing disordered spontaneous insertion. When proteoliposomes were completely free of detergent, MtlA was not integrated into (proteo) liposomes containing either SecYEG, YidC or MPIase. On the other hand, MtlA was efficiently integrated into proteoliposomes containing both SecYEG/MPIase. Furthermore, when the MtlA level was increased, YidC significantly enhanced the integration activity (Sasaki et al., 2019). From these results, we conclude that MPIase is also essential for Sec-dependent membrane integration, and that YidC promotes the MPIase-dependent integration in both the Sec-dependent and Sec-independent pathways.

MPIase also stimulates preprotein translocation of Sec-dependent presecretory protein, pOmpA (Moser et al., 2013). Many of the secretory proteins that function in the periplasmic region and outer membranes of *E. coli* are synthesized in the cytoplasm and are translocated through the SecYEG translocon on the inner membrane, and the preprotein translocation reaction on SecYEG is driven by a translocation ATPase, SecA (du Plessis et al., 2011) (Figure 1A4). Biochemical analysis suggests that SecA drives the preprotein translocation as follows. When SecA binds to a substrate protein on SecYEG, it undergoes a conformational change and is inserted deeply into SecYEG with the substrate protein (Economou and Wickner, 1994; du Plessis et al., 2011). Subsequently, ATP hydrolysis induces a conformational change and only SecA is released, leaving the substrate inside. This repetitive process is thought to drive the preprotein translocation in a stepwise manner (van der Wolk et al., 1997). It has also been shown that SecG, a component of SecYEG, undergoes the topology inversion cycle during preprotein translocation, facilitating the SecA cycle, and thereby the translocation activity (Nishiyama et al., 1996; Suzuki et al., 1998; Sugai et al., 2007). When MPIase was co-reconstituted with SecYEG, the translocation rate in the SecYEG-MPIase proteoliposomes was ∼10-fold accelerated compared with that in the SecYEG ones (Nishiyama et al., 2006; Moser et al., 2013). Similar results were obtained with the *in vivo* experimental system (Sawasato et al., 2019), suggesting that MPIase is also involved in preprotein translocation, and is expected to come into contact with SecYEG. Further analysis revealed that SecG inversion occurs only in the presence of MPIase (Moser et al., 2013), suggesting that direct interaction of MPIase with SecYEG enables SecG inversion and thus stimulates the translocation reaction. Indeed, MPIase transformed the SecYEG dimer from a “back-to-back” structure (Breyton et al., 2002; van den Berg et al., 2003) with SecE at the interface to a “side-by-side” structure with SecG in the proximity of the interface (Moser et al., 2013), demonstrating that MPIase affects the structural change of SecYEG including SecG inversion, and stimulates the preprotein translocation.

## Modes of action of DAG and membrane protein integrase in protein insertion

**FIGURE 2:**
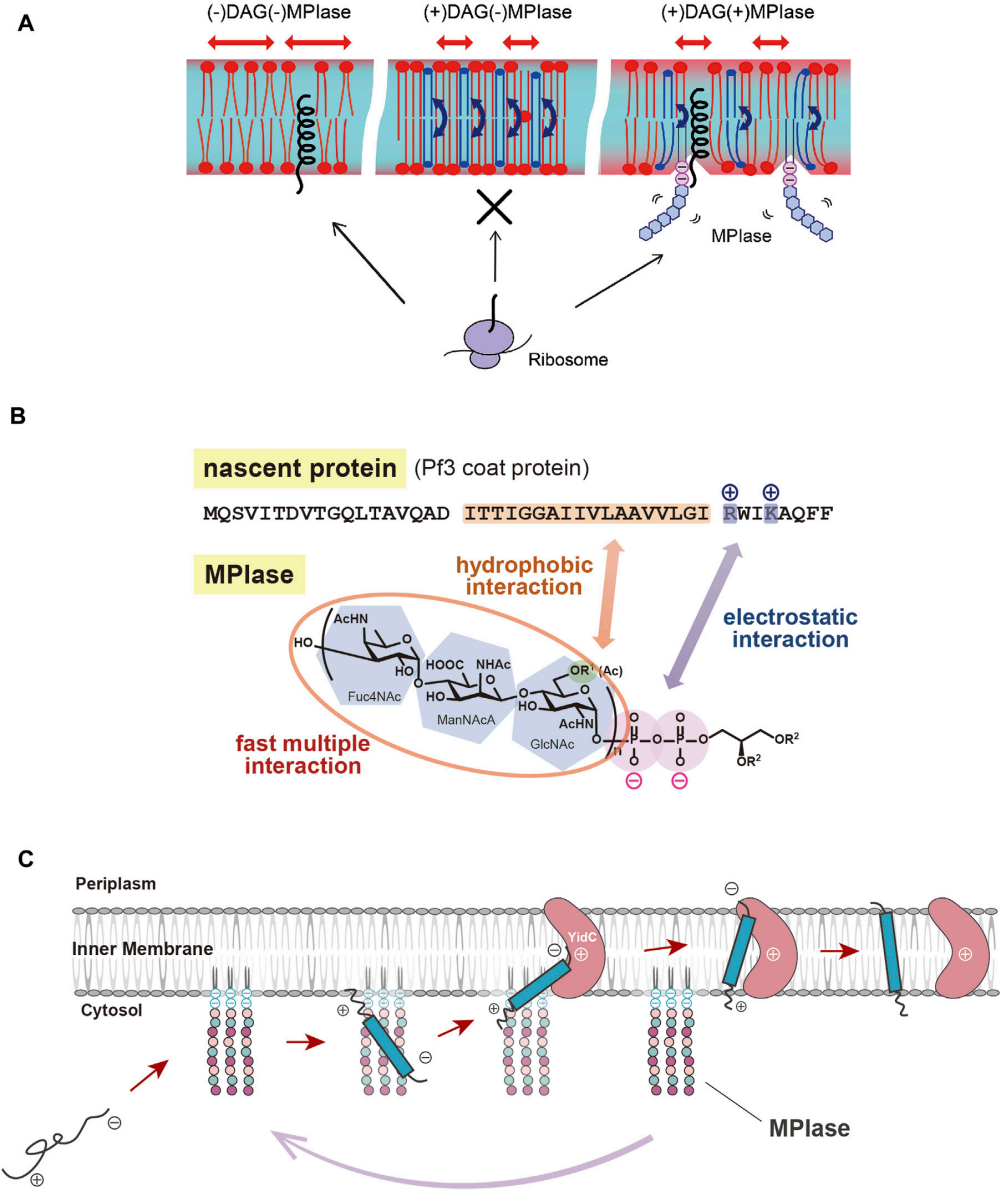
**(A)** Schematic diagram of the effects of DAG and MPIase on membrane insertion of substrate proteins. The blue arrows indicate the flip-flop motion of DAG. DAG is shown in blue and MPIase in purple (pyrophosphate) and light blue (glycan chain). **(B)** Model diagram of the interaction between the substrate protein (Pf3 coat) and MPIase that is thought to occur at the initial stage of membrane insertion in the *E. coli* inner membrane. The acetyl groups of MPIase and the TM region of the substrate protein cause hydrophobic interactions, while the pyrophosphate of MPIase and the basic amino acid residue of the protein cause electrostatic interactions. **(C)** Working model for cooperation of MPIase and YidC in the catalytic cycle in the Sec-independent membrane insertion.

As described above, spontaneous insertion is inhibited by physiological concentrations of DAG, while protein insertion proceeds through MPIase. PP-MPIase without the lipid moiety suppressed the aggregation of substrate proteins had lost the insertion activity (Fujikawa et al., 2018). Based on these findings, we speculated that DAG, an insertion inhibitor, and MPIase, a factor that promotes insertion, regulate the insertion reaction by altering the physicochemical properties of the membrane bilayers. We investigated the molecular mechanism of protein insertion by means of solid-state NMR and fluorescence measurements. The results indicated that DAG and MPIase regulate membrane lipid dynamics without disrupting the membrane structure integrity (Nomura et al., 2019). In the presence of DAG, the acyl groups of lipids are organized, and the membrane becomes packed; DAG quickly flip-flops, preventing lipids from diffusing laterally and filling the hydrophobic space formed by the acyl groups, thereby preventing proteins from entering the inside of the membrane from the cytosol. On the other hand, when MPIase is present in addition to DAG, the sugar chain of MPIase is thought to increase the motion of the membrane lipid headgroups, loosening the membrane packing and helping proteins associate with the membrane interior by disrupting the order of acyl groups in the membrane lipids and inhibiting the flip-flop motion of DAG (Nomura et al., 2019) (Figure 2A).

## Interaction between membrane protein integrase and substrate proteins

MPIase has been shown to be generally involved in membrane protein insertion by interacting with substrate proteins and membrane insertion factors such as YidC and SecYEG. Recently, the interaction between MPIase and a model substrate, Pf3 coat protein, was analyzed by physicochemical methods. It was shown that MPIase interacts with the TM region through the acetyl groups. Also, MPIase interacts with the basic residues on the C-terminal side of the substrates through the pyrophosphate moiety. Both hydrophobic and electrostatic interactions were found to occur simultaneously to promote membrane protein insertion, and possibly to determine the membrane topology (Mori et al., 2022) (Figure 2B). The interaction of MPIase with substrate proteins is transient and relatively nonspecific, suggesting that MPIase can facilitate the membrane insertion of a wide range of membrane proteins. The long sugar chains of MPIase were also found to allow the more rapid and repeated association and dissociation with the substrate. In the case of chemically synthesized mini-MPIase-3 (Figure 1B), of which membrane integration activity is low compared with that of MPIase, both the rates of association and dissociation with the substrates were slow, indicating that an appropriate length of the glycan chain is necessary for higher rates of association and dissociation with a substrate, which reflects the efficiency of membrane insertion (Mori et al., 2022). Mini-MPIase-3 in a membrane comprising *E. coli* lipids results in accelerated association/dissociation rates for substrates comparable to those of MPIase, suggesting that the clustering of trisaccharide units on the membrane plays a role like that of the long glycan chains of MPIase. The ability to rapidly associate and dissociate with substrates is consistent with the fact that MPIase can insert various types of substrate proteins into the membrane (Nishikawa et al., 2017; Sasaki et al., 2019; Sawasato et al., 2019; Endo et al., 2022), and with reports that MPIase repeats the membrane insertion cycle multiple times (Nishiyama et al., 2010).

## Future perspectives

For many years, membrane protein integration and preprotein translocation reactions have been thought to proceed through proteinaceous factors, but various biochemical, physicochemical and genetic analyses have revealed that the glycolipid MPIase is deeply involved in these reactions. MPIase localized on the membrane interacts with the substrate proteins through hydrophobic and electrostatic interactions via its long sugar chain and pyrophosphate moieties, preventing aggregation and leading a substrate to the membrane. The glycan moiety of MPIase causes protein insertion by loosening the membrane packing and delivering the substrate to the interior of the membrane. MPIase binds to factors such as YidC, which is also localized on the membrane, so that YidC receives the substrate proteins from MPIase. In this process, MPIase also functions in concert with other factors such as SecYEG and PMF, depending on the substrates’ hydrophobicity, charge distribution, expression level and so on. After releasing the substrate and passing it to another factor, MPIase binds to the next substrate protein and inserts it into the membrane (Figure 2C). Therefore, MPIase is “glycolipozyme” that catalyzes membrane protein insertion because it repeats this cycle multiple times.

It seems that mysteries and controversies on this field have been solved if the functions of MPIase are considered, as mentioned above. For example, the receptor and insertase for a subset of membrane proteins which have been thought to insert into membranes spontaneously had not been identified until MPIase was identified. The crystal structures of SecYEG did not explain the SecG inversion because of the artificial structures without MPIase. The insertase functions of YidC have not yet been fully understood in the absence of MPIase. From these, it is highly likely that MPIase directly interacts with the substrate membrane proteins, SecYEG and YidC. The mode of these interactions should be clarified in detail in near future.

Molecular mechanisms underlying protein integration and preprotein translocation reactions are important in biological phenomena that are conserved in all organisms from bacteria to higher eukaryotes at a fundamental level, and membrane integration factors such as SecYEG and YidC exist ubiquitously. Recently, an enzyme for MPIase biosynthesis in bacteria was identified (Sawasato et al., 2019). Homologues of this enzyme have also been found in yeast, human and chloroplasts, each of which has been shown to have the ability to biosynthesize MPIase (Sawasato et al., 2019; Sekiya et al., 2021). These results and a series of studies showing that MPIase is generally involved in protein transport strongly suggest the existence of MPIase homologs or factors with similar functions in eukaryotes. If eukaryotic MPIase homologs or analogs are found to exist, structure-function analyses of such factors will provide more detailed insights into the molecular mechanisms of protein transport. Moreover, functional modification of the MPIase homologues/analogues will enable the development of low-temperature tolerant plants, improvement of secretory production of useful proteins using microorganisms, or comprehensive functional analysis of membrane proteins as drug discovery targets.
